# Expansion of Psychosocial Care Centers and their relationship with health and human rights

**DOI:** 10.1590/0034-7167-2022-0662

**Published:** 2023-10-09

**Authors:** Antonio José Correa de Pauli, Caíque Rossi Baldassarini, Janaína Cristina Pasquini de Almeida, Jaqueline Lemos de Oliveira, Regina Célia Fiorati, Carla Aparecida Arena Ventura, Jacqueline de Souza

**Affiliations:** I Universidade de São Paulo. Ribeirão Preto, São Paulo, Brazil

**Keywords:** Mental Health Services, Primary Health Care, Human Rights, Cities, Health Systems., Servicios de Salud Mental, Atención Primaria de Salud, Derechos Humanos, Ciudades, Sistemas de Salud., Serviços de Saúde Mental, Atenção Primária à, Saúde, Direitos Humanos, Cidades, Sistemas de Saúde.

## Abstract

**Objective:**

To analyze the factors associated with the expansion of the number of Brazilian Psychosocial Care Centers (CAPS) considering aspects related to the general health scenario and the institutionalization of human rights.

**Methods:**

An analytical document-based study, developed between February 2020 and May 2022, whose secondary data on the 27 Brazilian capitals were collected on platforms in the public domain, based on the time series from 2015 to 2020. Indicators were listed based on health system infrastructure and quality of life. For data analysis, descriptive statistics, Pearson’s correlation test and Student’s t test were used.

**Results:**

The capitals that expanded the number of CAPS in the analyzed period were the ones that presented the greatest political-legal framework for the protection of human rights.

**Conclusion:**

The results suggest that the greater the commitment of governments in favor of human rights, the greater the investment for CAPS expansion.

## RESUMO

**Objetivo:**

Analisar os fatores associados à expansão do número de Centros de Atenção Psicossocial (CAPS) brasileiros considerando aspectos relacionados ao panorama geral da saúde e à institucionalização dos direitos humanos.

**Métodos:**

Estudo de base documental analítico, desenvolvido entre fevereiro de 2020 e maio de 2022, cujos dados secundários sobre as 27 capitais brasileiras foram coletados em plataformas de domínio público, fundamentando-se na série temporal de 2015 a 2020. Os indicadores foram elencados com base nos temas infraestrutura do sistema de saúde e qualidade de vida. Para a análise dos dados, empreenderam-se estatísticas descritivas, os Testes de Correlação de Pearson e t de Student.

**Resultados:**

As capitais que expandiram o número de CAPS no período analisado foram as que apresentaram maior arcabouço político-legal de proteção dos direitos humanos.

**Conclusão:**

Os resultados sugerem que, quanto maior o comprometimento dos governos em prol dos direitos humanos, maior o investimento para a expansão dos CAPS.

## RESUMEN

**Objetivo:**

Analizar los factores asociados a la expansión del número de Centros de Atención Psicosocial (CAPS) brasileños considerando aspectos relacionados con el panorama general de salud y la institucionalización de los derechos humanos.

**Métodos:**

Estudio analítico de base documental, desarrollado entre febrero de 2020 y mayo de 2022, cuyos datos secundarios sobre las 27 capitales brasileñas fueron recopilados en plataformas de dominio público, con base en la serie temporal de 2015 a 2020. Los indicadores fueron enumerados con base en los temas infraestructura del sistema de salud y calidad de vida. Para el análisis de los datos, se utilizó la estadística descriptiva, la prueba de correlación de Pearson y la prueba t de Student.

**Resultados:**

Las capitales que ampliaron el número de CAPS en el período analizado fueron las que presentaron mayor marco político-jurídico para la protección de los derechos humanos**. Conclusión:** Los resultados sugieren que cuanto mayor sea el compromiso de los gobiernos a favor de los derechos humanos, mayor será la inversión para la expansión de los CAPS.

## INTRODUCTION

The direction of a satisfactory Psychosocial Care Network (RAPS - *Rede de Atenção Psicossocial*) management permeates, among other factors, the investment that municipalities have made in the different points of care of the health network. Aspects such as network resources and texture, positioning of managers and professionals, as well as elements that are promising or barriers to the fulfillment of RAPS objectives and guidelines^(1-3)^ have been widely emphasized in discussions on the right to health and access to mental health care in Brazil. In this regard, the Psychosocial Care Centers (CAPS - *Centros de Atenção Psicossocial*), pioneers as extra-hospital devices in Brazilian mental health policies and the focus of the main investments regarding the psychosocial network implementation in the country in the years after Law 10,216/2001, stand out^(4)^.

There is in the literature a consolidated body of knowledge produced on the operation and assessment of such services in the different regions of the country, highlighting their importance for the mental health care psychosocial model expansion, including in terms of matrix support and articulation with Primary Care (PC)^(5-7)^. Despite this, a gap is identified in relation to studies that analyze the possible infrastructural, budget and human rights protection factors associated with the investment of municipal administrations in the expansion of these services in the country.

## OBJECTIVE

To analyze the factors associated with the expansion of the number of Brazilian CAPS considering aspects related to general health scenario and institutionalization of human rights.

## METHODS

### Ethical aspects

This study falls within the sole paragraph of Article 1 of Resolution 510/2016 of the Brazilian National Health Council^(8)^, which establishes specific ethical guidelines for the human and social sciences. Thus, the non-involvement of human beings in data collection waived the assessment by the REC/CONEP system.

### Study design, period and place

This is an analytical document-based study, developed from February 2020 to May 2022. It was decided to use 27 Brazilian capitals, as they constitute the main poles of socioeconomic, cultural development and health reference of their regions and states, also functioning as a link between the surrounding municipalities^(9-10)^. The study was guided based on the items described in the Meta-analyses of Observational Studies in Epidemiology (MOOSE) instrument, given its quality for synthesizing different data and results.

### Study protocol

The first step consisted of defining a framework for developing the empirical phase of the research. Based on this definition, the categories, the result indicators as well as the respective data sources were listed.

### Study development framework

ISO 37120^(11)^ and 37122^(12)^, Sustainable Cities Program^(13)^ and the Competitiveness Ranking of Municipalities were used as frameworks^(14)^. ISO 37120 is the first technical standard referring to sustainability in urban communities, as it defines methodologies for assessing the performance of urban services and quality of life^(11)^. Standard 37122 presents indicators to guide and assess the performance of urban services management and quality of life for smart cities^(12)^.

The Sustainable Cities Program is an urban sustainability agenda for Brazil that encompasses social, environmental, political, economic and cultural elements considered essential for municipal planning. This program is structured around 12 thematic axes that support the Sustainable Development Goals (SDGs), described in the 2030 Agenda for sustainable development, plans and goals for the planet^(13)^. Thus, it proposes indicators, tools and methodologies to support public management and urban planning. The Municipalities Competitiveness Ranking proposes 55 indicators related to institutions, society and economy, through which it assesses the competitiveness of municipalities with more than 80 thousand inhabitants^(14)^.

### Categories, outcome indicators and respective data sources

**Figure 1:**
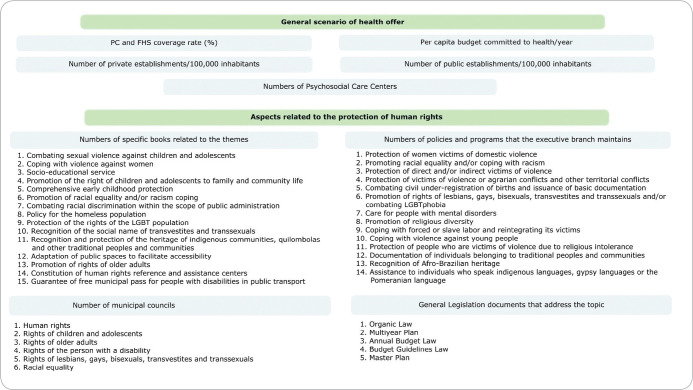
Categories, indicators and respective themes adopted for the study development, Brazil, 2022

Based on this documentary framework and considering the proposed objective, the main categories for selecting the indicators of this study were listed: general scenario of health offer and aspects related to protection of human rights. The indicators referring to each category as well as the themes considered in their unfolding are shown in Figure 1.

*Caption: in green are the categories; in blue, the indicators; and in blank and numbered, the topics listed by the Brazilian Institute of Geography and Statistics (Instituto Brasileiro de Geografia e Estatística) for assessing each of the respective indicators.*

Regarding the source of data for the second category indicators, specific modules from the Municipal Basic Information Survey (MUNIC - *Pesquisa de Informações Básicas Municipais*) from the Brazilian Institute of Geography and Statistics (IBGE - *Instituto Brasileiro de Geografia e Estatística*) were used^(15)^. For the first category indicators, four databases were used, namely: Primary Care Indicator Panels (SISAPS *- Painéis de Indicadores da Atenção Primária*)^(16)^, considering the PC coverage rate and the Family Health Strategy (FHS) for 2019; Municipal and State Public Finance System (FINBRA - *Sistema de Finanças Públicas Municipais e Estaduais*)^(17)^ (2019 budgets); Brazilian National Register of Health Establishments (CNES Data-SUS - *Cadastro Nacional de Estabelecimentos de Saúde*)^(18)^ for a survey of public and private establishments (2020); and CNESNet^(19)^ (old version of the Brazilian National Register of Health Establishments (*Cadastro Nacional de Estabelecimentos de Saúde*)) to identify the number of CAPS in the time series from 2015 to 2019.

### Database elaboration and collection procedures

Initially, a spreadsheet called “General Data” was created in Microsoft Excel 2019, in which the lines corresponded to each of the Brazilian capitals, and the columns, to the indicators adopted in the study. Information collection was carried out through online consultations on the public domain platforms described above.

The number of public, private and CAPS health establishments was consulted on the respective platforms, and such information was entered manually in the corresponding columns of the “General Data” worksheet. The percentage of PC coverage, FHS coverage, the number of budgets directed to health and all indicators related to human rights were obtained via direct download of the files made available in the aforementioned data sources. The data of interest from such files were copied and pasted in the columns assigned to them in the “General Data” worksheet.

Next, a new worksheet called “General Data 2” was prepared and the entire data collection procedure was performed again, which resulted in the construction of two similar data worksheets to check the values and identify possible errors. To do so, the “General Data” worksheet was copied and pasted onto “General Data 2” using the subtraction function. The resulting worksheet was named “Conference”. All cell values in the “Conference” worksheet should, therefore, be transformed into zero after subtraction. In those where this value was not identified, it was considered a possible error in data collection, so the information was again verified in the corresponding data source, and the entire process was repeated until an error-free worksheet was obtained.

### Analysis of results, and statistics

Data analysis comprised descriptive statistics, listing the quantitative variables’ percentages, means and standard deviation as well as Student’s t test to identify the difference in means obtained in the listed indicators, considering the capitals that expanded and those that did not expand the number of CAPS per inhabitant in the proposed time frame.

Specifically in relation to the protection of human rights, Pearson’s correlation test was carried out, taking into account the four listed indicators. As for this category, analysis was based on how the themes considered were presented in the constitution of councils, policies, programs and in the legal framework of such capitals. This option is based on the understanding that the more widely these topics are addressed, the more vulnerable situations and groups will be supported in institutional terms. For the interpretation of results, both the individual highlights and by regions (North, Northeast, Midwest, South and Southeast) were considered as well as the results of the statistical tests carried out.

## RESULTS

### General scenario of health offer

Most capitals presented budgets for the health sector in the range of R$500.00 (US$100.00) to R$900.00 (US$180.00) per person/year, and, although they predominantly had private health services, they also had good care coverage in PC and FHS. When analyzing the regional cut, the Midwest was the only region in which all capitals had PC and FHS coverage below 60%. The North and Northeast regions stood out in terms of low coverage in this regard, with the exception of Teresina (Northeast) and Palmas (North), which demarcated the existing contrast in the internality of these regions. It is also noteworthy that only six capitals from different regions had budgets greater than R$1,000.00 (US$200.00) per person/year, namely: Teresina (Northeast), Cuiabá, Campo Grande, Brasília (Midwest), Belo Horizonte (Southeast) and Porto Alegre (South) (Table 1).

**Table 1:** Distribution of capitals according to general health scenario and institutionalization of human rights, Brazil, 2021

Capital	Budget (R$ per capita)	Establishments/ 100 thousand inhabitants			Coverage (%)		Human rights			
		CAPS^^*^^	Public	Private	PC^†^	FHS^‡^	Councils	Policies and programs	General legislation	Specific laws
Aracaju	633	0.93	24	418	81	65	4	8	3	11
Belém	688	0.62	18	166	38	23	3	8	0	6
Belo Horizonte	1522	0.56	19	294	100	80	4	9	4	12
Boa Vista	633	0.87	47	119	57	49	2	1	4	1
Brasília	1069	0.43	17	276	53	54	6	12	4	9
Campo Grande	1555	0.69	23	230	52	63	4	5	5	7
Cuiabá	1232	0.85	31	327	52	54	5	3	1	14
Curitiba	972	0.72	13	415	48	33	4	8	5	5
Florianópolis	711	0.83	28	406	100	65	5	4	0	12
Fortaleza	865	0.69	11	248	61	58	5	3	1	3
Goiânia	889	0.65	17	264	59	44	6	12	4	9
João Pessoa	877	0.63	32	233	94	86	4	3	1	1
Macapá	326	0.64	26	86	55	49	4	4	2	2
Maceió	743	0.69	21	150	43	25	4	11	4	9
Manaus	419	0.20	23	72	52	46	4	8	3	2
Natal	843	0.57	24	240	55	48	3	2	3	7
Palmas	775	0.71	35	242	100	95	4	10	3	7
Porto Alegre	1010	1.00	26	373	70	48	5	8	2	14
Porto Velho	626	0.78	35	245	60	53	4	4	3	5
Recife	700	1.04	23	173	64	54	5	10	4	8
Rio Branco	325	0.43	61	169	59	53	4	6	5	9
Rio de Janeiro	696	0.52	11	252	51	40	4	8	4	11
Salvador	539	0.65	15	133	42	40	5	7	3	9
São Luís	855	0.55	19	127	44	38	4	3	1	5
São Paulo	909	0.74	12	208	63	40	6	9	3	11
Teresina	1302	0.82	24	131	100	100	4	12	1	11
Vitória	785	1.12	28	534	88	74	5	5	3	7

Note:

* Psychosocial Care Centers;

† Primary Care;

‡ Family Health Strategy.

Note: the graph scale ranges from 0 (center of the figure) to 0.000014 (outer edge), which corresponds to approximately 1.4 Psychosocial Care Centers per 100,000 inhabitants.

**Figure 2:**
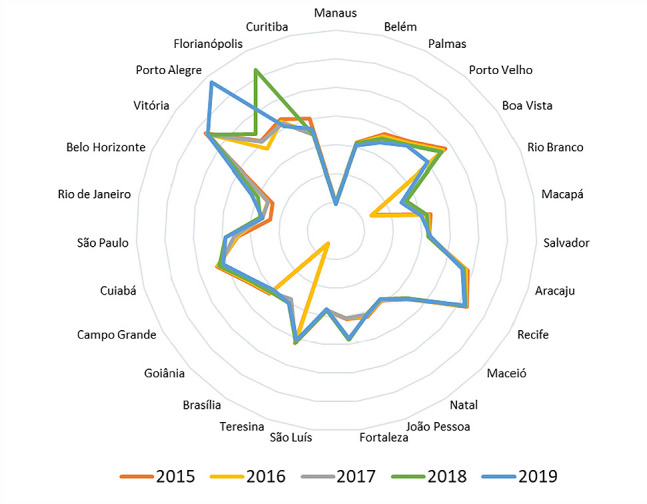
Variations in the number of Psychosocial Care Centers per capita in the period from 2015 to 2019, Brazil, 2021

Regarding the number of CAPS per inhabitant, as can be seen in Figure 2, most capitals maintained some stability over the analyzed time series. The expansion of these services was identified in the capitals Porto Alegre (South), Belo Horizonte, Rio de Janeiro, São Paulo (Southeast), Brasília (Midwest), Fortaleza, Salvador (Northeast) and Rio Branco (North).

### Human right protection

#### Councils, policies and programs, laws common to municipalities and specific laws

The cities of São Paulo, Brasília and Goiânia had six councils on human rights issues listed by the IBGE. The North region figured as the one that prioritized such mechanisms of popular participation on this issue to a lesser extent. Despite this, in most capitals (n=24), the councils covered at least three of these themes, as can be seen in Table 1.

Regarding policies and programs, none of the capitals covered the 14 possible themes (Table 1), which shows that in all regions there is some weakness in municipal action in relation to the social protection of different vulnerable groups. Despite this, all municipalities studied had at least one policy or program on human rights.

**Figure 3:**
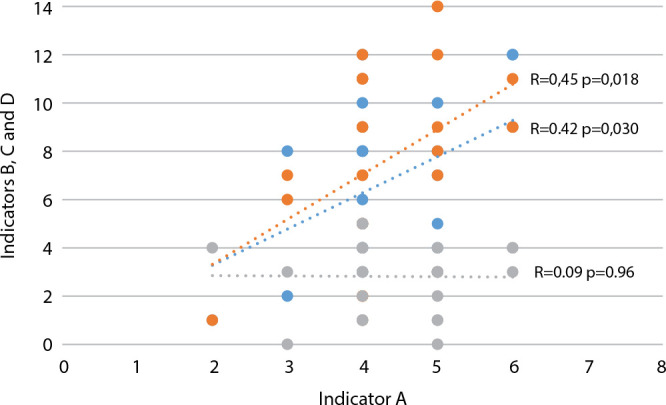
Correlation between the indicators adopted to analyze the human rights category, Brazil, 2021

With regard to general legislation, Curitiba (South), Rio Branco (North) and Campo Grande (Center-West) addressed the human rights issue in the five analyzed laws and there was no mention of the topic in any of these laws only in the records of Belém (North) and Florianópolis (South). The capitals contemplated, on average, eight of the total of 15 possible themes in specific laws, with emphasis on the Midwest, South and Southeast regions to the detriment of the North region, which presented the smallest legislative framework in terms of specific laws on the subject (average of the region=4.6). Despite this, all capitals had specific laws that covered at least one of the fifteen proposed themes. A positive correlation was identified between the variables “advices”, “policies and programs” and “specific laws”, as shown in Figure 3.

*Caption: indicators A) number of municipal councils related to the protection of human rights; B) number of policies and programs on this topic maintained by the executive branch (blue); C) number of laws within the scope of general legislation dealing with human rights (grey); D) number of specific laws related to human rights (orange).*

### Categories and the relationship with Psychosocial Care Center expansion

Considering the categories analyzed in the present study, it was identified that the capitals that expanded the number of CAPS in the period studied were also those that presented the highest average in relation to the number of councils, policies and programs and laws to protect human rights (Table 2).

**Table 2:** Difference in the average of general health scenario and institutionalization of human rights categories between Brazilian capitals that expanded (n=8) or not (n=19) the number of CAPS between 2015 and 2019, Brazil, 2021

Human rights and health scenario	Expanded	Did not expand	t	*p*
	Mean (SD^*^)	Mean (SD^*^)		
Institutionalization of HD^†^	25.63 (6.05)	19.89 (6.67)	2.09	0.047
Public health establishments	21.44 (16.85)	25.99 (7.81)	-0.97	0.341
Private health establishments	244.23 (75.00)	241.11 (127.79)	0.06	0.949
Health budget	866.78 (363.11)	819.12 (292.52)	0.36	0.722
PC coverage^‡^	0.62 (0.17)	0.65 (0.21)	-0.35	0.732
FHS coverage^§^	0.52 (0.14)	0.56 (0.21)	-0.50	0.625

Note:

* Standard Deviation;

† Human rights;

‡ Primary Care;

§ Family Health Strategy.

## DISCUSSION

The scenario obtained in the present study points to capitals in general, with a budget volume that exceeds the average of Brazilian municipalities (R$430.12 [US$ 86,02] per capita/year)^(20)^. Despite this, such a budget corresponds to less than R$2.20 (US$0.44) per day and, given that internationally people spend an average of 10 to 25% and, in Brazil, up to 40% of their income on health^(21-22)^, it is inferred that public investment in this regard is still below expectations in the studied capitals, with repercussions on the Brazilian scenario. Furthermore, this fact reiterates the international recommendations to increase public spending on health and prioritize PC coverage expansion, due to the direct implications in improving life expectancy and optimizing resources^(21)^.

In this regard, it is worth mentioning that the set of capitals presented, in a way, good PC and FHS coverage. However, the focus of the analysis on regional scenarios highlights the intense inequality existing in the country both in terms of resources and in the level of action of public health management and social development, aspects already widely described in the literature^(23-25)^.

Despite the current goal of expanding PC coverage globally^(21,26-27)^, several official reports from national and international health agencies also indicate the scarcity of specialized resources in mental health (services and professionals) and the existing gap between needs and resources in this area^(28-32)^. In this regard, the present study identified that few capitals expanded the number of CAPS over the analyzed time series, which was also described in recent studies on specialized and community-based mental health services in lowand middle-income countries^(33-34)^.

When analyzing these two aspects, PC coverage and availability of specialized mental health services, it is important to point out the strong national and international health agencies recommendations on the insertion of mental health actions in the scope of PC with a view to optimizing resources and reducing the gap in access to care in this area^(30-31,35)^. It is understood that such an initiative promotes assistance based more on an integral perspective, in addition to contributing to reduction of stigma related to mental disorders and suffering^(33)^.

Several recent studies have been developed on this topic, reaffirming the strategic potential of PC as a point of care in the psychosocial network, but also pointing out numerous barriers to the consolidation of such a proposal^(29,36)^. Issues such as the limits of the teams’ operational capacity and each network service’s technical skills are key points in this discussion, which also reiterates the importance of investments in the quality and coverage of specialized mental health services, which is a prerogative that corroborates the international goals and recommendations for such service qualification^(31,35)^. It is worth remembering that the target audience of CAPS consists of individuals with severe and persistent mental disorders^(29,37)^ and that prioritization of investments should be given to community-based services to the detriment of hospital services^(29)^.

In this way, the legitimacy of CAPS therapeutic projects stands out, which, through their specialized interdisciplinary teams, play a crucial role in proposing collaborative actions and articulation with the other health network and social protection device services^(29,37)^. In other words, the nature of a community-based service highlights it in terms of potential integration with the enrolled population, consolidation of intersectoral actions in the territory covered and implementation of actions to combat stigma^(29,36-37)^. This profile is similar to that of PC services, however psychosocial rehabilitation, as a theoretical and technical basis, is CAPS’ main specificity^(29)^.

Social reintegration and the rescue of citizenship, especially for patients discharged from long periods of hospitalization, operate a clear connection between the areas of mental health, law and social protection^(29,37)^. Thus, the result that pointed to a better political-legal framework for the protection of human rights in the capitals that expanded the number of CAPS in the analyzed period reiterates the solid body of ethical, political and technical discussions that emphasize mental health as an important part of the global human rights agenda as well as the strong commitment of the psychosocial model to such an agenda^(38)^.

On the other hand, the results of this study also express a scenario that lacks investments in legal terms and expansion of popular participation mechanisms, including in some regions of the country that illustrate a frank picture of inequalities as a striking aspect and that still persists in the national scope^(25)^.

Furthermore, the positive correlation identified between the number of specific councils and the number of human rights policies and programs suggests that spaces for active social participation positively influence the creation of more public actions to deal with this issue as well as possibly greater attribution of goals and resources to combat vulnerabilities.

In short, the results of this study demonstrate that the documentary framework that guides municipal managers’ actions has the potential to be reflected in an important way in the technical and operational scope^(38)^, which emphasizes the importance of popular participation in the formulation of policies locations^(39)^.

Furthermore, the inclusion of human rights in the legal framework aligned with PC coverage expansion, improved access to quality mental health services, in addition to policies and programs to combat stigma and discrimination, was reaffirmed in the present study and also appear as the main international health and social protection agencies recommendations^(31,35)^.

It appears, therefore, that the greater the commitment of the country, state, city in guaranteeing such rights to society, the greater the commitment/investment in proposals that favor freedom, acceptance and respect for diversity, including in the field of health mental.

### Limitations of the study

As limitations of this study, firstly, there is the fact that only the number of CAPS was adopted as the main coverage indicator. It is understood that the use of other indicators, such as quality, resolution and access to these services, would provide more robust results. Furthermore, data related to the demand for such mental health services would also broaden the spectrum of analysis, since many individuals with severe and persistent disorders may be being treated in other services, such as PC, general hospitals or emergency services. Secondly, there is a lack of uniformity in the dates of all the databases used due to gap in updating them by the respective public bodies. It is understood that this reflects, in a way, the country’s low investment in the generation’s assiduity and availability of public data on official platforms, which compromises a more effective assessment of the country’s public policies. However, it is understood that these limits do not make the results and discussions presented less relevant, as they provide concrete subsidies for reflection and decision-making in relation to the theme and proposed approach.

### Contributions to nursing, health, or public policies

This study contributes to a better understanding of the association between the offer of RAPS devices, in particular CAPS, and the general scenario of health and the institutionalization of human rights in Brazilian capitals, municipalities that act as a reference in different levels of health care. Based on the results obtained, which suggest a positive relationship between the expansion of the main RAPS mental health service (CAPS) and the institutionalization of human rights in laws and government actions, its importance is noted, since it guides new studies on the subject and reinforces that achieving human rights in the spheres of the executive and local legislature can interfere with the development of a mental health service’s infrastructure in the city.

## CONCLUSIONS

The factors associated with the increase in mental health units based on the psychosocial care model (CAPS) were those related to the greater presence of human rights in public spaces, i.e., the capitals that had more councils, policies, programs and specific laws to deal with the protection and public actions related to human rights were more successful in terms of increasing the supply of units of this service for their population.

It was identified that, in the analyzed period, less than half of capitals increased the number of CAPS per inhabitants, a result that does not corroborate the official reports, which, when considering the regions and states as a whole, indicate a scenario of frank expansion in the country. In addition to this, CAPS reflect only a portion of the psychosocial care offered and, as they are considered pioneering services in the psychosocial network, the fact that they have low coverage indirectly reveals a certain precariousness in relation to other extra-hospital devices in different regions of the country.

On the other hand, this low expansion in the number of specialized services may indicate that other care scenarios are absorbing the demand of individuals with severe and persistent mental disorders. Therefore, it is suggested that future studies consider indicators related to the demands for mental health care in all health care and social protection devices.

## Data Availability

https://doi.org/10.48331/scielodata.R3KUZK

## References

[1] Medrado ACC, Cruz MG, Baião JJ, Souza MA, Araújo PS. (2018). Os laços e nós de uma rede de atenção psicossocial. Cad Bras Saúde Mental.

[2] Querino RA, Borges RS, Almeida LY, Oliveira JL, Souza J. (2020). Psychosocial care network: perception of managers and tensioning of field. Rev Bras Enferm.

[3] Sampaio ML, Bispo JP. (2021). Network of Psychosocial Care: evaluation of the structure and process of mental healthcare linkage. Cad Saúde Pública.

[4] Presidência da República (BR) (2001). Dispõe sobre a proteção e os direitos das pessoas portadoras de transtornos mentais e redireciona o modelo assistencial em saúde mental.

[5] Iglesias A, Avellar LZ. (2019). Matrix support in mental health: practices and concepts brought by reference teams, matrix teams and managers. Cien Saude Colet.

[6] Silva SN, Lima MG, Ruas CM. (2018). Brazilian Mental Health Services Assessment: user satisfaction and associated factors. Cien Saude Colet.

[7] Souza IJ, Weber L, Lopes SM, Colussi CF, Nickel DA. (2021). Avaliação de Serviços de Atenção Psicossocial no Brasil: Uma revisão integrativa de literatura. Braz J Mental Health.

[8] Ministério da Saúde (BR) (2016). Conselho Nacional de Saúde. Resolução nº 510, de 07 de abril de 2016. Dispõe sobre as normas aplicáveis a pesquisas em Ciências Humanas e Sociais.

[9] Instituto Brasileiro de Geografia e Estatística (IBGE) (2022). Estimativas da População.

[10] Instituto Brasileiro de Geografia e Estatística (IBGE) (2022). REGIC - Regiões de Influência das Cidades.

[11] Associação Brasileira de Normas Técnicas (2017). NBR ISO 37120: desenvolvimento sustentável de comunidades: indicadores para serviços urbanos e qualidade de vida.

[12] Associação Brasileira de Normas Técnicas (2020). NBR ISO 37122: cidades e comunidades sustentáveis - Indicadores para cidades inteligentes.

[13] Instituto Cidades Sustentáveis (ICS) (2022). Programa Cidades Sustentáveis.

[14] Centro de Liderança Pública (2022). Ranking de Competitividade dos Municípios.

[15] Instituto Brasileiro de Geografia e Estatística (IBGE) (2019). MUNIC - Pesquisa de Informações Básicas Municipais.

[16] Ministério da Saúde (BR) (2022). Painéis de Indicadores da Atenção Primária.

[17] Tesouro Nacional (BR) (2020). Área Publica. Sistema de Informações Contábeis e Fiscais do Setor Público Brasileiro (Siconfi). Contas Anuais 2015-2020.

[18] Ministério da Saúde (BR) (2019). Cadastro Nacional de Estabelecimentos de Saúde (CNES Data-SUS).

[19] Ministério da Saúde (BR) (2019). Cadastro Nacional de Estabelecimentos de Saúde (CNESNet).

[20] Conselho Federal de Medicina (CFM) (2019). Brasil gasta R$ 3,83 ao dia com a saúde de cada habitante.

[21] World Health Organization (WHO) (2019). Executive summary.

[22] Moraes RM, Santos MAB, Werneck HF, Paula MN, Almeida RT. (2022). Gastos das famílias com planos de saúde no Brasil e comprometimento da renda domiciliar: uma análise da Pesquisa de Orçamentos Familiares (2017/2018). Cad Saúde Pública.

[23] Silva FF, Gomes AM, Barbosa A, Lucena WGL. (2020). Explanatory factors of the economic efficiency of the public expenditure with health of the Brazilian capital cities. Rev Científ Hermes.

[24] Guimarães WSG, Parente RCP, Guimarães TLF, Garnelo L. (2018). Acesso e qualidade da atenção pré-natal na Estratégia Saúde da Família: infraestrutura, cuidado e gestão. Cad Saúde Pública.

[25] Garnelo L, Lima JG, Rocha ESC, Herkrath FJ. (2018). Access and coverage of Primary Health Care for rural and urban populations in the northern region of Brazil. Saúde Debate.

[26] Pan American Health Organization (PAHO) (2019). APS Para la salud universal OPS/OMS.

[27] Global Burden of Disease Health Financing Collaborator Network (2018). Trends in future health financing and coverage: future health spending and universal health coverage in 188 countries, 2016-40. Lancet.

[28] Pheister M, Cowley D, Sanders W, Keeble T, Lu F, Pershern L (2022). Growing the Psychiatry Workforce Through Expansion or Creation of Residencies and Fellowships: the Results of a Survey by the AADPRT Workforce Task Force. Acad Psychiatry.

[29] Pinho ES, Souza ACS, Esperidião E. (2018). Working processes of professionals at Psychosocial Care Centers (CAPS): an integrative review. Cien Saude Colet.

[30] Jetty A, Petterson S, Westfall JM, Jabbarpour Y. (2021). Assessing Primary Care Contributions to Behavioral Health: a Cross-sectional Study Using Medical Expenditure Panel Survey. J Prim Care Community Health.

[31] Patel V, Saxena S, Lund C, Thornicroft G, Baingana F, Bolton P (2018). The Lancet Commission on global mental health and sustainable development. Lancet.

[32] World Health Organization (WHO) (2020). Mental Health Atlas.

[33] Kohrt BA, Asher L, Bhardwaj A, Fazel M, Jordans MJD, Mutamba BB (2018). The Role of Communities in Mental Health Care in Lowand Middle-Income Countries: a meta-review of components and competencies. Int J Environ Res Public Health.

[34] Roberts T, Esponda GM, Krupchanka D, Shidhaye R, Patel V, Rathod S. (2018). Factors associated with health service utilisation for common mental disorders: a systematic review. BMC Psychiatr.

[35] World Health Organization (WHO) (2019). Special initiative for mental health (2019-2023).

[36] Onocko-Campos RT, Amaral CEM, Saraceno B, Oliveira BDC, Treichel CAS, Delgado PGG. (2018). Atuação dos Centros de Atenção Psicossocial em quatro centros urbanos no Brasil. Rev Panam Salud Publica.

[37] Bongiovanni J, Silva RAN. (2019). Desafios da desinstitucionalização no contexto dos serviços substitutivos de saúde mental. Psicol Soc.

[38] Duffy RM, Kelly BD. (2017). Rights, laws and tensions: a comparative analysis of the Convention on the Rights of Persons with Disabilities and the WHO Resource Book on Mental Health, Human Rights and Legislation. Int J Priv Health Inf Manag.

[39] Guarnieri JM, Setti SM, Pulga VL. (2021). A participação popular na saúde: desafios e potencialidades no contexto municipal. Rev Saúde Redes.

